# The Effect of Age on Improvement in Health‐Related Quality of Life After Percutaneous Coronary Intervention

**DOI:** 10.1002/clc.70260

**Published:** 2026-01-20

**Authors:** Laura Lappalainen, Piia Lavikainen, Risto P. Roine, Harri Sintonen, Janne Martikainen, Anna‐Maija Tolppanen, Juha Hartikainen

**Affiliations:** ^1^ Kuopio University Hospital Kuopio Finland; ^2^ University of Eastern Finland Kuopio Finland; ^3^ University of Helsinki, Helsinki Finland and Kuopio University Hospital Kuopio Finland; ^4^ University of Helsinki Helsinki Finland; ^5^ Kuopio University Hospital, Kuopio Finland and University of Eastern Finland Kuopio Finland

**Keywords:** coronary artery disease, health‐related quality of life, patient‐reported outcomes, Percutaneous coronary intervention

## Abstract

**Introduction:**

Percutaneous coronary intervention (PCI) is the first‐line therapy in patients scheduled for coronary revascularization, aiming to relieve symptoms of coronary artery disease (CAD) and improve health‐related quality of life (HRQoL) and prognosis. Particularly, in older adults, symptom alleviation and HRQoL are emphasized. However, it is not known whether older patients benefit from PCI equally to their younger peers. We used disease‐specific and generic instruments to evaluate the improvement in HRQoL after PCI, comparing changes in three age groups.

**Methods:**

Altogether 300 patients undergoing PCI were divided into three age groups: ≥ 75 years (*n* = 89), 66–74 years (*n* = 117), and ≤ 65 years (*n* = 94). HRQoL was measured using the disease‐specific Seattle Angina Questionnaire (SAQ‐7) and the generic 15D instrument at baseline, one, and 12 months.

**Results:**

Statistically and clinically significant improvements in the SAQ‐7 and 15D scores were observed after one‐ and 12‐month follow‐up in all age groups. There were no differences in the 12‐month improvements in the SAQ‐7 and 15D scores between the groups. The 15D score started to decline after 1 month, particularly in the oldest group. The decline was associated with age‐related rather than CAD‐related 15D dimensions.

**Conclusions:**

Our findings on comparable improvement in disease‐specific and generic HRQoL after PCI in older and younger patients are encouraging, particularly considering that the aims of PCI in older adults are predominantly symptom alleviation and improvement of daily activities. In addition, to overcome age‐related changes in HRQoL, a disease‐specific instrument should be incorporated in the evaluation of PCI on HRQoL.

**Clinical trial registration:**

5101114.

## Graphical abstract

The effect of age on improvement in health‐related quality of life (HRQoL) after percutaneous coronary intervention (PCI)—Do older adults benefit from PCI equally to their younger peers in respect of HRQoL and symptom relief?

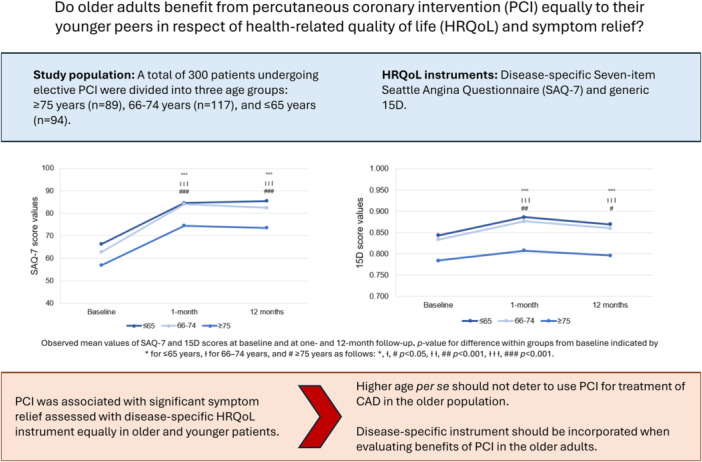

## 1 Introduction

Coronary artery disease (CAD) is a chronic, usually progressive disease caused by atherosclerotic plaques accumulating in the epicardial coronary arteries. The key to chronic CAD treatment is optimal medical therapy (OMT), aiming to reduce symptoms and prevent the progression of CAD and atherothrombotic events. If adequate symptom control is not achieved with OMT and the patient has a manifestation of myocardial ischemia, revascularization (percutaneous coronary intervention, PCI, or coronary artery bypass grafting, CABG) is justifiable [1].

PCI aims to relieve symptoms caused by CAD and improve health‐related quality of life (HRQoL) and to reduce the risk of cardiac adverse events and death [1]. In addition, in younger patients, the goal is to improve life expectancy and disease prognosis, while among older patients, symptom alleviation and maintenance of functional ability are emphasized [2].

PCI has become the first‐line therapy in patients with elective and emergency revascularizations [3], and it is performed increasingly in the aged population. Old age is an essential non‐modifiable risk factor and predictor of complications such as bleeding, renal failure, and neurological complications in patients undergoing revascularization [1, 4]. In addition, old patients have a higher prevalence of comorbidities and more complex CAD compared to the young [5]. Advanced age is associated with worse short‐ and long‐term outcome after PCI [5, 6]. Moreover, the aged population is underrepresented in clinical trial studies [1, 7], and thereby it is not well known whether the old patients benefit from PCI equally to their younger peers with respect to symptom relief and HRQoL. Previous studies have evaluated the HRQoL after PCI in the older population [8, 9, 10, 11, 12, 13, 14, 15, 16, 17, 18, 19, 20, 21, 22, 23], but comparison between older and younger patients has not been made [8, 9, 10, 15, 21], or assessed instruments have been only either generic [8, 9, 10, 14, 15, 16, 20, 22, 23] or disease‐specific [11, 12]. Moreover, some studies have combined patients undergoing elective and urgent PCI [15, 16, 20, 22] and different revascularization modalities (PCI and CABG) [8, 9, 10, 12, 13] or evaluated only very specific patient groups such as chronic total occlusion [17]. In addition, it has not been well established whether disease‐specific instruments provide valuable additional information that they should be incorporated in the evaluation of treatment effects on HRQoL. To address these knowledge gaps, we compared the changes in disease‐specific and generic HRQoL after elective PCI between older (age ≥ 75 years) and younger patients (66–74 years and ≤65 years). HRQoL was assessed with the disease‐specific seven‐item Seattle Angina questionnaire (SAQ‐7) [24] and generic quality of life (15D) instruments [25].

## 2 Materials and Methods

### 2.1 Study Protocol

Altogether 335 stable patients with chronic CAD undergoing elective PCI between May 2017 and January 2019 at Kuopio University Hospital Heart Center were recruited in this prospective cohort study. The only exclusion criterion was unwillingness or inability to participate in the study or to give informed consent.

PCI was performed according to local practice. Periprocedural and postprocedural medication was based on the operators' direction. Follow‐up time was 12 months.

The study complied with the Declaration of Helsinki statement, and the local Ethics Committee of Northern Savo approved the study. All patients gave informed written consent to participate in the study, and participation did not affect their treatment.

### 2.2 Outcomes

The primary outcomes of this study were in HRQoL measured by patient‐assessed disease‐specific SAQ‐7 [24] and generic 15D instruments [25]. HRQoL of the patients was measured prior to the PCI and at one and 12 months thereafter.

SAQ‐7 is a validated disease‐specific instrument measuring the perspectives of CAD patients' health status with a 4‐week recall period. It comprises three domains of health: physical limitation (SAQ‐PL), angina frequency (SAQ‐AF), and quality of life (SAQ‐QL). The SAQ‐PL consists of three questions, while the SAQ‐AF and SAQ‐QL comprise two questions each. The SAQ‐7 summary score is calculated as the mean of domain scores, and it represents the health status of CAD patients, with 0 denoting the worst and 100 the best health status. In the SAQ‐7 summary score, a change of 5−8 points is considered clinically significant [24].

15D questionnaire measures generic HRQoL, using fifteen dimensions of health (“mobility,” “vision,” “hearing,” “breathing,” “sleeping,” “eating,” “speech,” “excretion,” “usual activities,” “mental function”, “discomfort and symptoms,” depression,” “distress,” “vitality,” and “sexual activity”) [26]. Each dimension has five options to choose from to describe respondents' current health status. The single index score (15D score), representing the overall HRQoL on a scale of 0–1 (1 = full health, 0 = being dead) and the dimension level values, reflecting goodness of the levels relative to no problems on the dimension (=1) and to being dead (=0), are calculated by using population‐based preference or utility weights [25]. Based on respondents' other answers, age, and gender, up to three missing answers can be imputed by using an algorithm‐based regression method with a multiple imputation procedure to replace the missing data [26]. A change of ±0.015 in the 15D score is considered clinically important [27].

### 2.3 Statistical Analysis

Patients were divided into three age groups (≥75, 66–74, and ≤65 years). The baseline characteristics of the age groups are shown as mean (standard deviation, SD) or frequency (percentages). Between‐group differences in clinical characteristics were tested with one‐way ANOVA with LSD post hoc tests (normally distributed), or with Kruskal−Wallis test and Mann−Whitney test for pairwise comparisons (skewed distribution). Differences in categorical variables between the groups were tested with the Pearson Chi‐square or Fisher's Exact test.

An unadjusted repeated measures linear mixed model was used to examine the changes in HRQoL 1 and 12 months after PCI within the age groups, assuming an unstructured covariance matrix. In addition, linear mixed models with variance components were used to assess the difference in the 12‐month improvement of HRQoL between the age groups, adjusting for baseline HRQoL and characteristics that differed between the groups at baseline with *p* < 0.05. Additionally, we compared the 12‐month improvements in SAQ‐7 and 15D scores after adjustment for comorbidity burden: hypertension, atrial fibrillation, diabetes, prior myocardial infarction, prior revascularization, heart valve stenosis or regurgitation, heart failure, prior stroke or transient ischemic attack, existing peripheral artery disease, chronic obstructive lung disease, current smoking, or kidney failure (glomerulus filtration rate < 60 mL/min/1.73 m^2^) [28, 29] by using linear mixed model with variance components. HRQoL values are denoted as mean (95% confidence interval, CI).

## 3 Results

**Figure 1:**
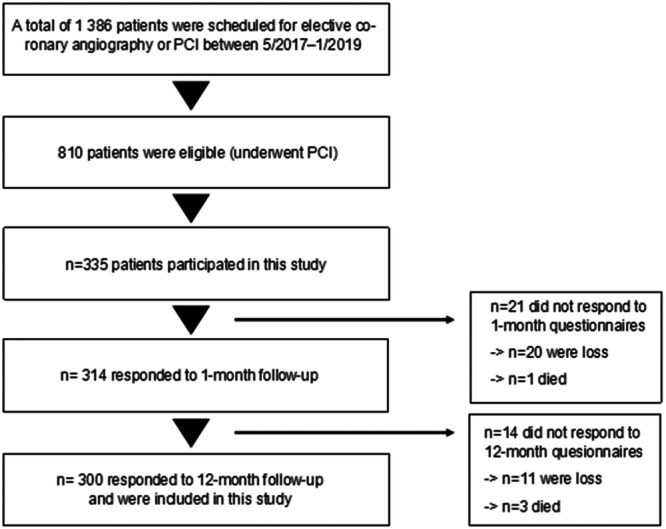
Study flow chart.

A total of 300 (89.6%) of the 335 recruited patients responded to the baseline, 1‐, and 12‐month follow‐up questionnaires and were included in the study (Figure 1). Four of the nonrespondents (1.2% of recruited patients) died during the follow‐up. The clinical characteristics of included and excluded patients within age groups were very comparable (Supporting Information S1: Table 1). Only, history of previous myocardial infarction was more common in the excluded patients than among the included patients (40.0% vs. 19.3% *p* = 0.008).

The final study population (*n* = 300) were divided into three age groups; 89 patients aged ≥ 75 years, 117 patients aged 66–74, and 94 patients aged ≤ 65 (Table 1). The median ages in the three age groups were 79, 69, and 60 years, and the proportions of women 36.0%, 32.5%, and 19.1%, respectively. At baseline, atrial fibrillation, a history of stroke or transient ischemic attack, and kidney failure were more common and Canadian Cardiovascular Score (CCS) was higher in the oldest age group compared with the two youngest groups. In addition, compared to the younger groups, patients ≥ 75 years were more often women, had more commonly history of CABG, left main disease, and valve disease whereas they were less often smokers and had lower prevalence of hypercholesterolemia, obesity, and family history of CAD. Among older adults, comorbidity burden was significantly higher (*p* = 0.002) compared to the younger groups. Adverse outcomes during 12‐month follow‐up were very rare (21.3%, 23.1%, and 21.3% in patients ≥ 75 years, 66–74 years, and ≤65 years, respectively) without differences between groups (*p* = 0.441) (Supporting Information S1: Table 2).

**Table 1:** Clinical characteristics of study population at baseline.

	≤ 65 (*n* = 94)	66–74 (*n* = 117)	≥ 75 (*n* = 89)	*p*	Post hoc analysis		
					≤ 65 versus 66–74	≤ 65 versus ≥ 75	66–74 versus ≥ 75
Age	60 (56, 62)	69 (67, 71)	79 (77, 83)	< 0.001	< 0.001	< 0.001	< 0.001
Sex, women	18 (19.1)	38 (32.5)	32 (36.0)	0.028	0.041	0.013	0.657
Hypertension	73 (77.7)	99 (84.6)	72 (80.9)	0.432	0.215	0.716	0.575
Hyperkolesterolemia	93 (98.9)	109 (93.2)	79 (88.8)	0.018	0.045	0.004	0.323
Diabetes mellitus	25 (26.6)	40 (34.2)	26 (29.2)	0.473	0.294	0.743	0.547
Obesity (BMI > 30 kg/m^2^)	38 (41.8)	33 (28.9)	16 (19.8)	0.007	0.076	0.003	0.180
Family History	55 (59.8)	55 (47.4)	35 (40.2)	0.029	0.093	0.011	0.321
Current smoker	12 (13.8)	9 (7.7)	3 (3.4)	0.036	0.176	0.017	0.239
Atrial fibrillation	8 (8.5)	15 (12.8)	30 (33.7)	< 0.001	0.378	< 0.001	< 0.001
Previous CAD	56 (59.6)	70 (59.8)	64 (71.9)	0.135	1.000	0.088	0.078
Previous MI	12 (12.8)	25 (21.4)	21 (23.6)	0.139	0.144	0.082	0.737
Previous PCI	28 (29.8)	43 (36.8)	37 (41.6)	0.246	0.308	0.122	0.564
Previous CABG	7 (7.4)	13 (11.1)	18 (20.2)	0.028	0.480	0.017	0.079
PAD	4 (4.3)	9 (7.7)	9 (10.1)	0.310	0.393	0.155	0.622
Stroke/TIA	6 (6.4)	5 (4.3)	15 (16.9)	0.004	0.545	0.036	0.004
Valve stenosis/regurgitation	3 (3.2)	14 (12.0)	14 (15.7)	0.038	0.040	0.012	0.539
COPD/asthma	20 (21.3)	13 (11.1)	19 (21.3)	0.075	0.056	1.000	0.053
Kidney failure (GFR < 60 mL/min/1.73m2)	0 (0.0)	8 (6.9)	20 (22.7)	< 0.001	0.004	< 0.001	0.002
LVEF	59.8 (11.2)	58.3 (10.9)	57.9 (9.8)	0.391	0.288	0.201	0.647
Heart failure (LVEF < 40%)	4 (4.3)	8 (6.8)	4 (4.5)	0.649	0.554	1.000	0.559
Diseased vessels				0.297	0.039	0.371	0.614
1‐VD	36 (38.3)	30 (25.6)	28 (31.5)				
2‐VD	33 (35.1)	44 (37.6)	29 (32.6)				
3‐VD	25 (26.6)	43 (36.8)	32 (36.0)				
LM disease	7 (7.4)	15 (12.8)	17 (19.1)	0.064	0.259	0.027	0.247
CCS				0.007	0.580	0.002	0.024
1	3 (3.2)	3 (2.6)	1 (1.1)				
2	64 (68.8)	70 (60.9)	39 (43.8)				
3	24 (25.8)	40 (34.8)	42 (47.2)				
4	2 (2.2)	2 (1.7)	7 (7.9)				

*Note:* The values denote mean and interquartile ranges (age) or standard deviation (LVEF) or *n* (%). *p* denotes the statistical significance between the age groups.

Abbreviations: BMI, body mass index; CABG, coronary artery bypass grafting; CAD, coronary artery disease; CCS, Canadian cardiovascular score (1 = “no angina in ordinary physical activities”, 2 = “slight limitation in ordinary physical activities”, 3 = “marked limitation in ordinary physical activities”, 4 = “inability to manage daily activities”); COPD, chronic obstructive pulmonary disease; GFR, glomerulus filtration rate; LM, left main; LVEF, left ventricular ejection fraction; MI, myocardial infarct; PAD, peripheral artery disease; PCI, percutaneous coronary intervention; TIA, transient ischemic attack; VD, vessel disease.

### 3.1 SAQ‐7 at Baseline, and at 1‐Month and 12‐Month Follow‐Up

**Figure 2:**
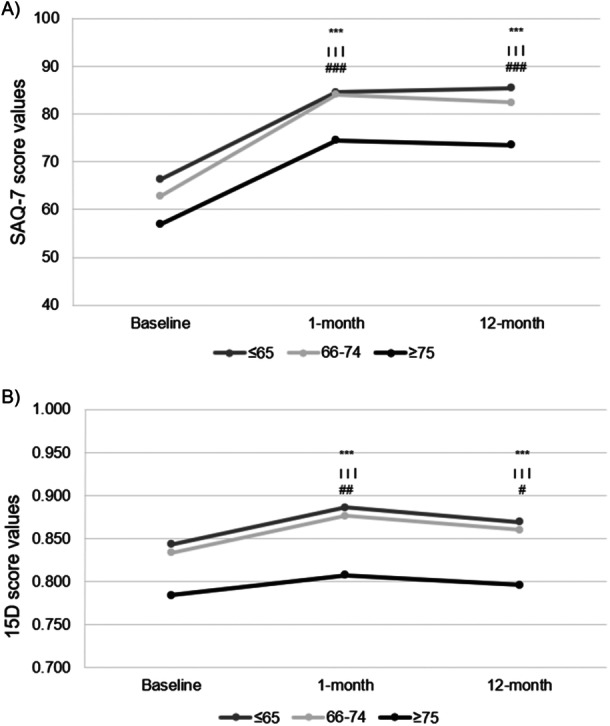
Observed mean values of (A) Seattle Angina Questionnaire short‐form (SAQ‐7) and (B) 15D scores at baseline and at 1‐ and 12‐month follow‐up. *p* value for difference from baseline is obtained with repeated measures linear mixed model and indicated by * for ≤ 65 years, ^Ɨ^ for 66–74 years, and ^#^ ≥ 75 years as follows: *, ^Ɨ^, ^#^ *p* < 0.05, **, ^Ɨ Ɨ^, ^##^ *p* < 0.01, and ***, ^Ɨ Ɨ Ɨ^, ^###^ *p* < 0.001.

At baseline, SAQ‐7 score was significantly lower in the oldest group (mean 56.9, 95% CI 52.7–61.1) compared to the midmost group (mean 62.8, 95% CI 58.8–66.8) and the youngest group (mean 66.3, 95% CI 62.3–70.3) (Figure 2a, Table 2). SAQ‐PL measuring physical limitations was significantly higher in the youngest age group compared to the two older groups (*p* < 0.001). On the other hand, SAQ‐7 domains measuring angina frequency (SAQ‐AF) and quality of life (SAQ‐QL) showed no significant difference between the age groups.

**Table 2:** Seattle Angina Questionnaire short‐form (SAQ‐7) at baseline and changes after one‐ and 12‐month follow‐up.

	Baseline scores			*p*	1‐Month change			12‐Month change		
	≤ 65	66–74	≥ 75		≤ 65	66–74	≥ 75	≤ 65	66–74	≥ 75
SAQ‐7	66.3 (62.3–70.3)	62.8 (58.8–66.8)	56.9 (52.7–61.1)	0.008	18.1 (13.7–22.5)	21.2 (17.2–25.3)	17.4 (12.4–22.4)	18.7 (14.4–23.1)	19.3 (15.2–23.5)	16.0 (11.9–20.0)
SAQ‐PL	84.0 (80.2–87.7)	76.9 72.7–81.1)	64.1 (58.6–69.6)	< 0.001	6.7 (3.2–10.1)	11.3 (6.8–15.9)	13.9 (8.8–18.9)	4.6 (0.2–9.0)	8.9 (5.2–12.6)	6.0 (1.1–11.0)
SAQ‐AF	66.5 (61.0–71.9)	62.9 (58.1–67.7)	60.3 (55.3–65.4)	0.272	19.8 (14.1–25.5)	23.8 (18.6–29.1)	18.3 (12.7–23.8)	21.5 (16.4–26.7)	21.2 (15.9–26.5)	16.0 (11.0–20.9)
SAQ‐QL	49.3 (43.8–54.8)	48.9 (43.8–54.1)	47.4 (41.7–53.2)	0.884	28.2 (22.0–34.5)	29.5 (24.0–34.9)	21.6 (14.3–28.9)	31.9 (25.1–37.9)	28.4 (22.4–34.3)	22.8 (16.5–29.0)

*Note: p* values denote the statistical significance of the difference in means between the age groups at baseline by using a repeated measures linear mixed model. The values denote means (95% confidence intervals).

Abbreviations: SAQ‐AF, SAQ Angina Frequency; SAQ‐PL, SAQ Physical limitations; SAQ‐QL, SAQ Quality of life.

All age groups improved their mean SAQ‐7 summary score both statistically and clinically significantly at 1‐ and 12‐month follow‐up (Figure 2a, Table 2). In addition, compared to baseline, the mean improvement of SAQ‐7 summary score showed no difference between the age groups at one or 12 months (Supporting Information S1: Table 3). By adjusting the SAQ‐7 score with covariates including in comorbidity burden, the change at 12 months was in the oldest group 15.5 (95% CI 5.3–25.7), in the midmost group 18.0 (7.1–28.9), and the youngest group 15.6 (4.3–26.9), showing no differences between the groups (*p* = 0.657).

**Figure 3:**
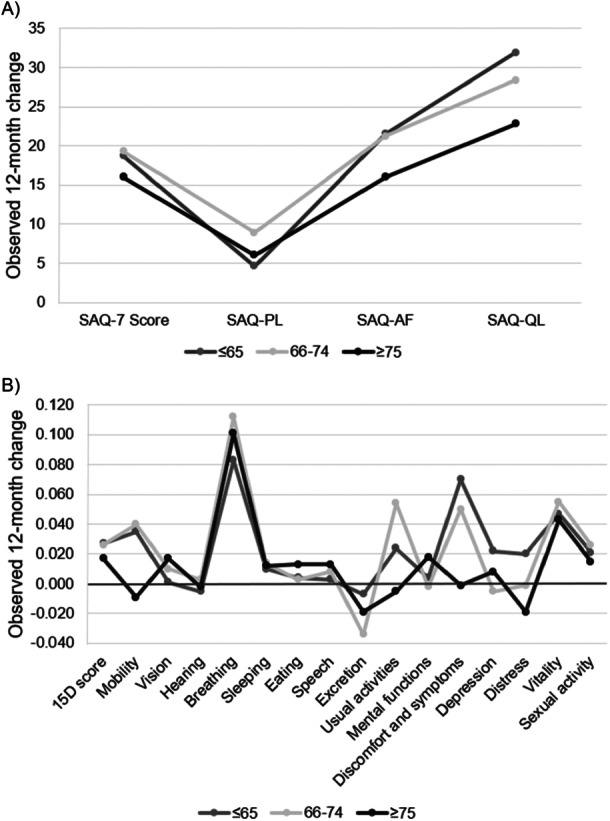
Observed changes in (A) SAQ‐7 score and SAQ‐7 domains, physical limitations (SAQ‐PL), angina frequency (SAQ‐AF), and quality of life (SAQ‐QL), and (B) 15D score and 15D dimensions at 12‐month follow‐up.

Statistically significant mean improvements were observed in SAQ‐7 dimensions (SAQ‐AF, SAQ‐QL, and SAQ‐PL) in all age groups after one‐ and 12‐month follow‐up (Figure 3a, Table 2). The 12‐month mean improvements in SAQ‐AF and SAQ‐QL showed no significant difference between the groups (Supporting Information S1: Table 3). However, there was no improvement in the SAQ‐PL domain in the two youngest age groups, and a decrease of 4.2 points was observed in the oldest age group, resulting in a significant between‐group difference (*p* = 0.005).

### 3.2 15D at Baseline and at 1‐ and 12‐month Follow‐Up

The baseline 15D score was significantly lower in the oldest group (mean 0.784, 95% CI 0.762–0.806) than in patients aged 66–74 years (mean 0.833, 95% CI 0.815–0.852) and patients aged 65 or younger (mean 0.843, 95% CI 0.832–0.863) (Figure 2b, Table 3). Particularly, the mean scores for several dimensions (“vision,” “hearing,” “breathing,” “speech,” “excretion,” “usual activities,” “mental functions,” “vitality,” and “sexual activity”) were lower in the oldest age group compared to the two younger groups.

**Table 3:** 15D at baseline and changes at 1‐ and 12‐month follow‐up.

	Baseline scores			*p*	1‐Month change			12‐Month change		
	≤ 65	66–74	≥ 75		≤ 65	66–74	≥ 75	≤ 65	66–74	≥ 75
15D Score	0.843 (0.823–0.863)	0.833 (0.815–0.852)	0.784 (0.762–0.806)	< 0.001	0.041 (0.028–0.054)	0.042 (0.029–0.055)	0.024 (0.010–0.040)	0.025 (0.012–0.042)	0.026 (0.013–0.038)	0.017 (0.001–0.032)
Mobility	0.846 (0.813–0.878)	0.836 (0.805–0.867)	0.791 (0.751–0.831)	0.126	0.063 (0.032–0.094)	0.053 (0.022–0.084)	0.032 (−0.005–0.070)	0.035 (−0.003–0.072)	0.040 (0.009–0.070)	−0.009 (−0.049–0.031)
Vision	0.935 (0.907–0.963)	0.947 (0.927–0.968)	0.878 (0.837–0.920)	0.016	0.006 (−0.019–0.031)	0.002 (−0.016–0.019)	0.028 (−0.009–0.065)	0.001 (−0.025–0.016)	0.010 (−0.011–0.031)	0.017 (−0.026–0.060)
Hearing	0.938 (0.909–0.966)	0.934 (0.909–0.959)	0.807 (0.761–0.852)	< 0.001	0.004 (−0.018–0.025)	0.003 (−0.008–0.014)	0.008 (−0.026–0.043)	−0.005 (−0.026–0.016)	0.003 (−0.003–0.009)	−0.002 (−0.038–0.034)
Breathing	0.747 (0.707–0.787)	0.667 (0.625–0.708)	0.593 (0.547–0.639)	< 0.001	0.119 (0.083–0.155)	0.114 (0.072–0.157)	0.090 (0.037–0.142)	0.083 (0.042–0.125)	0.112 (0.076–0.148)	0.101 (0.054–0.148)
Sleeping	0.745 (0.704–0.786)	0.794 (0.759–0.829)	0.777 (0.737–0.818)	0.175	0.024 (−0.009–0.056)	0.042 (0.016–0.068)	0.020 (−0.014–0.055)	0.010 (−0.021–0.004)	0.014 (−0.015–0.043)	0.012 (−0.019–0.043)
Eating	0.992 (0.981–1.000)	0.994 (0.985–1.000)	0.979 (0.961–0.997)	0.208	0.004 (−0.004–0.012)	0.003 (−0.008–0.014)	0.012 (−0.002–0.027)	x	0.003 (−0.003–0.009)	0.013 (−0.002–0.027)
Speech	0.972 (0.954–0.990)	0.964 (0.947–0.982)	0.924 (0.895–0.952)	0.008	0.016 (0.002–0.029)	0.008 (−0.004–0.019)	0.020 (−0.001–0.040)	0.003 (−0.016–0.022)	0.008 −0.006–0.021)	0.013 (−0.010–0.036)
Excretion	0.873 (0.838–0.909)	0.864 (0.830–0.897)	0.802 (0.760–0.844)	0.021	0.033 (0.001–0.065)	−0.010 (−0.034–0.013)	−0.003 (−0.038–0.032)	−0.007 (−0.050–0.036)	−0.034 (−0.062–0.006)	−0.019 (−0.062–0.024)
Usual activities	0.825 (0.785–0.865)	0.772 (0.730–0.814)	0.729 (0.682–0.776)	0.014	0.035 (0.000–0.070)	0.079 (0.044–0.114)	0.011 (−0.028–0.050)	0.024 (−0.014–0.063)	0.054 (0.022–0.086)	−0.005 (−0.050–0.041)
Mental functions	0.905 (0.873–0.938)	0.885 (0.854–0.916)	0.815 (0.774–0.856)	0.002	−0.004 (−0.031–0.024)	0.015 (−0.011–0.041)	0.019 (−0.022–0.060)	0.004 (−0.026–0.033)	−0.002 (−0.030–0.026)	0.018 (−0.022–0.059)
Discomfort and symptoms	0.663 (0.618–0.709)	0.672 (0.634–0.709)	0.646 (0.599–0.693)	0.690	0.072 (0.029–0.115)	0.092 (0.054–0.130)	0.038 (−0.008–0.085)	0.070 (0.029–0.111)	0.050 (0.010–0.089)	−0.001 (−0.052–0.051)
Depression	0.875 (0.842–0.907)	0.920 (0.898–0.943)	0.881 (0.851–0.910)	0.045	0.016 (−0.014–0.046)	0.014 (−0.005–0.034)	−0.003 (−0.032–0.026)	0.022 (−0.008–0.052)	−0.005 (−0.029–0.020)	0.008 (−0.020–0.036)
Distress	0.836 (0.801–0.871)	0.905 (0.879–0.943)	0.883 (0.850–0.916)	0.006	0.033 (0.007–0.059)	0.002 (−0.020–0.025)	−0.026 (−0.058–0.007)	0.020 (−0.009–0.048)	−0.001 (−0.029–0.026)	−0.019 (−0.047–0.010)
Vitality	0.759 (0.725–0.792)	0.745 (0.713–0.778)	0.700 (0.671–0.728)	0.022	0.080 (0.050–0.110)	0.075 (0.044–0.106)	0.044 (0.014–0.074)	0.047 (0.015–0.079)	0.055 (0.029–0.082)	0.043 (0.015–0.070)
Sexual activity	0.749 (0.692–0.805)	0.678 (0.625–0.731)	0.603 (0.535–0.672)	0.007	0.046 (0.004–0.088)	0.043 (0.009–0.077)	0.001 (−0.043–0.040)	0.021 (−0.024–0.066)	0.026 (−0.012–0.064)	0.015 (−0.035–0.066)

*Note: p* denotes the statistical significance of the difference between the age groups at baseline obtained by repeated‐measure linear mixed model. The values denote means (95% confidence intervals). x = cannot be computed; the standard error of the difference is 0.

Statistically and clinically significant improvement in the mean 15D score at 1‐month follow‐up was observed in all age groups (Figure 2b, Table 3). Thereafter 15D score started to decline, but nevertheless, statistically and clinically significant improvements were observed in all groups at 12‐months compared to baseline. In addition, the improvement in 15D score at 12 months did not differ between the age groups (*p* = 0.092) (Supporting Information S1: Table 3). In the comorbidity‐adjusted analysis, the 12‐month change was 0.015 (95% CI −0.018 to 0.048) in the oldest group, 0.028 (95% CI −0.007 to 0.063) in the group aged 66–74 years, and 0.024 (95% CI −0.013 to 0.061) in the youngest age group (*p* = 0.461).

Compared to baseline, mean 15D dimension scores of “breathing” and “vitality” improved statistically significantly in all age groups at 1‐ and 12‐month follow‐up (Figure 3b, Table 3). Additionally, dimensions “discomfort and symptoms” in the two youngest groups and dimension “usual activities” in the middlemost group remained improved at 12 months. At 12‐month follow‐up, the change in dimensions “mobility” (*p* = 0.016), “discomfort and symptoms” (*p* = 0.025), and “sexual activity” (*p* = 0.005) showed significant between‐group differences favoring the youngest age group (Supporting Information S1: Table 3).

## 4 Discussion

The main findings of our study were that compared to the baseline, the disease‐specific SAQ‐7 summary score improved clinically and statistically significantly, and its domains SAQ‐AF and SAQ‐QL improved statistically significantly at one and 12 months in all age groups without a significant difference between the groups. Clinically and statistically significant improvement in the generic HRQoL 15D score, as well as statistically significant improvement on 15D dimensions “breathing” and “vitality” at 1‐ and 12 months after PCI was observed in all age groups showing no difference between the groups. However, 15D score started to decline between 1‐and 12‐month follow‐up, particularly in the oldest group. This was driven by dimensions associated with ageing rather than CAD (“mobility,” “vision,” and “excretion”).

PCI is currently the first‐line invasive therapy in patients with drug‐refractory CAD. In addition, due to an aging population, the number of older patients undergoing PCI is rising. Old age is associated with comorbidities, increased risk of procedure‐related complications, and worse long‐term outcomes following PCI. In light of this, it is surprising that there is a paucity of evidence on whether the improvements of HRQoL after PCI differ between the old and the young patients. Agarwal et al. [14] studied HRQoL after elective PCI on octogenarians and concluded that both disease‐specific (SAQ) and generic (SF‐36) HRQoL improved after PCI but remained lower compared to the age‐matched general population. No comparison between older and younger patients was done. Chait et al. [15] evaluated the effect of PCI on generic HRQoL (SF‐36) in nonagenarians and demonstrated a HRQoL improvement similar to that of the general population corrected for age and sex. However, a great majority of patients (94%) suffered from acute coronary syndrome, and comparison between older and younger patients was not addressed. Moreover, the effect of different revascularization modalities (PCI and CABG) on HRQoL has been studied in the aged population [8, 9, 10, 11, 12], but the treatment outcomes have not been compared to younger patients, and patients undergoing PCI and CABG have not been studied separately. The elegant study by Seto et al. [18] examined changes in HRQoL among older patients after PCI and compared the changes with younger patients. However, the data was collected between 1994 and 1995, before the era of drug‐eluting stents and more advanced PCI techniques.

In our study, patients ≥ 75 years had a lower SAQ‐7 summary score compared to the two younger groups at baseline. However, the SAQ‐7 summary score improved both statistically and clinically significantly at 1‐month and remained improved at 12‐month follow‐up in all age groups. Importantly, the improvement showed no significant difference between the older and the younger patients. Recently, the ISCHEMIA trial, a landmark study comparing invasive and conservative treatment strategies in CAD, reported that the invasive strategy improved SAQ summary score at 12 months in all age groups, however, favoring the invasive strategy for the younger patients. What has to be taken into account is that in the ISCHEMIA trial invasive strategy included both revascularization modalities (PCI 74% and CABG 26%) [13]. Thus, any solid conclusions about the effects of PCI cannot be drawn.

Except for the ISCHEMIA trial, the SAQ‐7 summary score has not been used to examine the effect of PCI on HRQoL in aged populations. However, SAQ‐7 has been addressed in two studies by using SAQ‐7 domains, SAQ‐PL, SAQ‐AF, and SAQ‐QL. Seto et al. [18] reported a 12‐month improvement in SAQ‐AF in 75% and 74% of patients aged ≥ 70 years and those aged < 70 years, respectively. In addition, the improvement did not differ between the older and younger patients. In accordance with Seto et al. [18], in our study, SAQ‐AF and SAQ‐QL improved in all age groups without significant between‐group differences. As discussed above, the study by Seto et al. [18] was performed before the currently used modern PCI techniques such as drug‐eluting stents, drug‐eluting balloons, and advanced techniques. Correspondingly, in a recent study by Zhao et al. [17] encompassing 1076 patients aged < 65 years, 65–75 years, and >75 years undergoing PCI for chronic total occlusion, SAQ‐AF improved at 1 and 12 months equally in all age groups. However, because chronic total occlusion is a highly selective population of PCI patients, the result may have limited generalisability to the general PCI population.

SAQ‐PL improved at 1 month in all age groups. Thereafter, it began to decline in all groups, and particularly in the oldest patients, SAQ‐PL was lower compared to the two younger age groups at 12 months. This is in line with Zhao et al. [17] who reported improvement on SAQ‐PL at 1 month, whereas at 12 months SAQ‐PL was lower in the oldest patients compared to the younger groups [17].

In our study, the mean 15D score improved at 1‐month in all age groups, the change showing no differences between the groups. After 1 month, 15D score started to decline but persisted clinically significant at 12 months in all age groups. Using the same age groups as we did, Yan et al. [16] reported improvement in EQ‐5D VAS index score, and Zhao et al. [17] improvement in SF‐12 score after PCI in all age groups at 12 months after elective PCI. However, as mentioned above, it must be taken into consideration that Zhao et al. [17] studied patients undergoing revascularization of chronic total occlusion, and in the study by Yan et al. [16], both elective and acute CAD patients were included.

Improvement in the 15D score was predominantly attributable to improvements on dimensions of “breathing” and “vitality.” The change in these dimensions has been reported to capture the disease‐specific information on SAQ‐PL [30]. The improvement in “breathing” and “vitality” is also in accordance with earlier studies reporting that the gain in HRQoL was predominantly due to improvement in physiological functioning scores in EQ‐5D, SF‐12, and SF‐36 instruments [18, 19, 21].

Of note, in our study, the mean 15D dimension scores of “mobility,” “vision,” and “excretion” in the oldest age group decreased during the follow‐up, which most likely reflects age‐related changes and are unlikely to be associated with treatment outcomes of PCI. Yan et al. [16] also reported greater deterioration in mobility, self‐care, and usual activities beyond 6 months after PCI among the older patients.

The strengths of our study are low exclusion criteria and high response rates for avoiding the bias of missing data [31]. Moreover, the evidence of similarity between included and excluded patients reduces the selection bias. However, we also acknowledge potential limitations of our study. This is single site study, and the number of patients in different age groups is limited, which impacts the statistical power for between‐group comparisons. However, the main results, that is, the post‐procedural improvement in HRQoL in the older population was evident despite the limited sample size. Although information on a range of comorbidities was obtained from patients' records, we did not have more detailed information on frailty, which is common among older CAD patients [32]. Moreover, only one patient was diagnosed with dementia. Thus, our results may not be useful when assessing the treatment strategy in patients with frailty and dementia.

## 5 Conclusion

PCI was associated with significant symptom relief assessed with a disease‐specific HRQoL instrument (SAQ‐7), both in the older and younger patients after 1‐ and 12‐month follow‐ups. Generic HRQoL (15D) also improved at 1 month, started to decline thereafter, but remained improved compared to baseline at 12‐month follow‐up in all age groups. The improvement in the mean 15D was driven by improvements on physical functioning dimensions (“breathing” and “vitality”), whereas the decline after 1 month was due to age‐related dimensions (“mobility,” “vision,” and “excretion”) rather than dimensions associated with the severity of CAD. Our findings on comparable improvement in disease‐specific and generic HRQoL after PCI in older and younger patients are encouraging, particularly considering that the aims of PCI in older adults are predominantly symptom alleviation and improvement of daily activities. Therefore, higher age *per se* should not deter to use PCI for the treatment of CAD in older adults. In addition, our study suggests that to overcome age‐related changes in HRQoL, a disease‐specific instrument should be incorporated when evaluating the possible benefits of PCI on HRQoL.

## Ethics Statement

All patients gave informed written consent to participate in the study. The study complied with the Declaration of Helsinki statement, and the local Ethics Committee of Northern Savo approved the study (approval number 135/2017).

## Consent

All patients gave informed written consent to participate in this study.

## Conflicts of Interest

A.‐M.T. acknowledges a research grant from Amgen, paid to the institution where she is employed (outside of the submitted work). J.M. is a founding partner of ESiOR Oy. This company was not involved in carrying out this research. H.S. is the developer of the 15D and obtains royalties from its electronic versions. The other authors declare no conflicts of interest.

## Supporting information


**Supplementary Table 1:** Clinical characteristics of excluded patients by age groups. **Supplementary Table 2:** Clinical events between groups during the 12‐month follow‐up. **Supplementary Table 3:** Estimated marginal means for 12‐month changes in SAQ‐7 and 15D instruments.

## Data Availability

The data generated and analyzed in the current study are not available due to participants' privacy, but are available from the corresponding author on reasonable request.
