# Supplementary material for: The Effect of Age on Improvement in Health‐Related Quality of Life After Percutaneous Coronary Intervention
Source: Clin Cardiol. 2026 Jan 20;49(1):e70260. doi: 10.1002/clc.70260 (PMC12817284; doi:10.1002/clc.70260)
Supplement: Supplementary file 1 — Supplementary Table 1: Clinical characteristics of excluded patients by age groups. Supplementary Table 2: Clinical events between groups during the 12‐month follow‐up. Supplementary Table 3: Estimated marginal means for 12‐month changes in SAQ‐7 and 15D instruments. [file CLC-49-e70260-s001.docx]

**Supplementary Table 1: Clinical characteristics of excluded patients by age groups**

|  | **≤65 (n=12)** | **66–74 (n=11)** | **≥75 (n=12)** | ***p*** |
| --- | --- | --- | --- | --- |
|  |  |  |  |  |
| **Age** | 55.8±6.5 | 69.1±2.2 | 78.8±4.0 | 0.366 |
| **Sex, women** | 3 (25.0) | 3 (27.3) | 3 (25.0) | 0.844 |
| **Hypertension** | 11 (91.7) | 3 (81.8) | 11 (91.7) | 0.358 |
| **Hyperkolesterolemia** | 10 (83.3) | 10 (90.9) | 11 (91.7) | 0.479 |
| **Diabetes mellitus** | 3 (25.0) | 3 (27.3) | 8 (66.7) | 0.252 |
| **Obesity**  **(BMI >30 kg/m^2^)** | 5 (41.7) | 2 (20.0) | 4 (36.4) | 0.696 |
| **Family Burden** | 8 (66.7) | 6 (54.5) | 4 (33.3) | 0.859 |
| **Current smoker** | 3 (25.0) | 1 (9.1) | 1 (8.3) | 0.222 |
| **Atrial fibrillation** | 0 (0.0) | 3 (27.3) | 4 (33.2) | 0.815 |
| **Previous CAD** | 10 (83.3) | 8 (72.7) | 9 (75.0) | 0.135 |
| **Previous MI** | 4 (33.3) | 4 (36.4) | 6 (50.0) | 0.008 |
| **Previous PCI** | 7 (58.3) | 5 (45.5) | 6 (50.0) | 0.096 |
| **Previous CABG** | 0 (0.0) | 1 (9.1) | 2 (16.7) | 0.597 |
| **PAD** | 1 (8.3) | 2 (18.2) | 1 (8.3) | 0.332 |
| **Stroke/TIA** | 0 (0.0) | 1 (9.1) | 2 (16.7) | 1.000 |
| **Valve stenosis/regurgitation** | 0 (0.0) | 0 (0.0) | 1 (8.3) | 0.227 |
| **COPD/asthma** | 1 (8.3) | 0 (0.0) | 5 (41.7) | 1.000 |
| **Kidney failure (GFR <60 ml/min/1.73m2)** | 0 (0.0) | 1 (9.1) | 3 (25.0) | 0.762 |
| **LVEF** | 55.0±6.1 | 55.5±18.9 | 50.4±18.0 | 0.149 |
| **Heart failure (EF <40%)** | 0 (0.0) | 1 (9.1) | 2 (16.7) | 0.433 |
| **Diseased vessels** |  |  |  | 0.658 |
| **1-VD** | 2 (16.7) | 6 (54.5) | 4 (33.3) |  |
| **2-VD** | 9 (75.0) | 4 (36.4) | 1 (8.3) |  |
| **3-VD** | 1 (8.3) | 1 (9.1) | 7 (58.3) |  |
| **LM** | 3 (25.0) | 1 (54.5) | 1 (8.3) | 0.793 |
| **CCS** |  |  |  | 0.138 |
| **1** | 1 (8.3) | 0 (0.0) | 0 (0.0) |  |
| **2** | 4 (33.3) | 8 (72.7) | 3 (25.0) |  |
| **3** | 7 (58.3) | 3 (27.3) | 9 (75.0) |  |
| **4** | 0 (0.0) | 0 (0.0) | 0 (0.0) |  |

Abbreviations: BMI= body mass index, CABG=coronary artery bypass grafting, CAD= coronary artery disease, CCS= Canadian cardiovascular score (1=”no angina in ordinary physical activities”, 2=”slight limitation in ordinary physical activities”, 3=”marked limitation in ordinary physical activities”, 4=”inability to manage daily activities”), COPD= chronic obstructive pulmonary disease, GFR= glomerulus filtration rate, LVEF= left ventricular ejection fraction, MI= myocardial infarct, PAD= peripheral artery disease, PCI= percutaneous coronary intervention, VD= vessel disease. The values denote mean and interquartile ranges (age) or standard deviation (LVEF) or n (%). *p* denotes the comparison between included and excluded patients.

**Supplementary Table 2: Clinical events between groups during the 12-month follow-up**

|  | **≤65 years (n=94)** | **66–74 years (n=117)** | **≥75 years (n=89)** | ***p*** |
| --- | --- | --- | --- | --- |
| **All** | 20 (21.3) | 27 (23.1) | 19 (21.3) | 0.441 |
| **Death** | 1 (1.1) | 2 (1.7) | 1 (1.1) | 0.659 |
| **MI** | 3 (3.2) | 5 (4.3) | 3 (3.4) | 0.906 |
| **PCI** | 8 (8.5) | 15 (12.8) | 6 (6.7) | 0.509 |
| **CABG** | 0 (0.0) | 0 (0.0) | 0 (0.0) | – |
| **Major bleeding** | 6 (6.4) | 3 (2.6) | 6 (6.7) | 0.371 |
| **Stroke/TIA** | 2 (2.1) | 2 (1.7) | 3 (3.4) | 0.718 |

Abbreviations: CABG= coronary artery bypass grafting, PCI=percutaneous coronary intervention, MI= myocardial infarction, TIA= transient ischemic attack. *p* denotes the statistical significance between age groups.

**Supplementary Table 3: Estimated marginal means for 12-month changes in SAQ-7 and 15D instruments.**

|  | **Multivariate model^a^** | | | |
| --- | --- | --- | --- | --- |
|  | **≤65** | **66–74** | **≥75** | ***p*** |
|  |  |  |  |  |
| **SAQ-7 score** | 14.8  (9.4–20.2) | 13.9  (8.8–19.1) | 8.4  (3.5–13.3) | 0.057 |
| **SAQ-PL** | 4.0  (-1.4–9.4) | 4.5  (-0.7–9.8) | -4.2  (-9.4–1.0) | 0.005 |
| **SAQ-AF** | 16.4  (10.5–22.4) | 14.9  (9.3–20.6) | 10.2  (4.9–15.6) | 0.135 |
| **SAQ-QL** | 23.8  (16.3–31.4) | 20.8  (13.6–28.0) | 15.9  (9.2–22.7) | 0.146 |
|  |  |  |  |  |
| **15D Score** | 0.030  (0.009–0.051) | 0.025  (0.005–0.045) | 0.006  (-0.015–0.026) | 0.092 |
| **Mobility** | 0.005  (-0.042–0.052) | -0.002  (-0.046–0.043) | -0.061  (-0.104­–[-0.018]) | 0.016 |
| **Vision** | -0.036  (-0.072–0.000) | -0.019  (-0.053–0.015) | -0.037  (-0.069–[-0.005]) | 0.485 |
| **Hearing** | -0.007  (-0.020–0.007) | -0.004  (-0.016–0.008) | 0.003  (-0.009–0.016) | 0.409 |
| **Breathing** | 0.106  (0.052–0.160) | 0.095  (0.044–0.146) | 0.066  (0.015–0.116) | 0.378 |
| **Sleeping** | -0.022  (-0.067–0.023) | 0.002  (-0.040–0.043) | 0.000  (-0.040–0.039) | 0.473 |
| **Eating** | -0.001  (-0.012–0.009) | 0.002  (-0.007–0.012) | 0.007  (-0.002–0.016) | 0.330 |
| **Speech** | 0.093  (-0.096–0.282) | -0.017  (-0.193–0.160) | -0.027  (-0.196–0.142) | 0.370 |
| **Excretion** | -0.020  (-0.071–0.032) | -0.054  (-0.103–[-0.006]) | -0.061  (-0.107–[-0.014]) | 0.240 |
| **Usual activities** | 0.017  (-0.034–0.068) | 0.016  (-0.033–0.065) | -0.035  (-0.082–0.012) | 0.089 |
| **Mental functions** | 0.025  (-0.020–0.069) | 0.004  (-0.037–0.046) | -0.020  (-0.060–0.020) | 0.170 |
| **Discomfort and symptoms** | 0.057  (-0.006–0.120) | 0.034  (-0.025–0.093) | -0.032  (-0.089–0.026) | 0.025 |
| **Depression** | 0.012  (-0.027–0.052) | 0.011  (-0.026–0.048) | 0.002  (-0.034–0.037) | 0.856 |
| **Distress** | -0.005  (-0.047–0.038) | -0.001  (-0.041–0.038) | -0.032  (-0.070–0.006) | 0.304 |
| **Vitality** | 0.043  (0.000–0.085) | 0.047  (0.007–0.087) | 0.026  (-0.013–0.066) | 0.602 |
| **Sexual activity** | 0.085  (0.023–0.147) | 0.036  (-0.022–0.095) | -0.027  (-0.085–0.031) | 0.005 |

^a^ Adjusted for BL scores, sex, and comorbidities: atrial fibrillation, obesity, previous revascularization, stroke/TIA

Abbreviations: SAQ-AF= SAQ Angina Frequency, SAQ-PL= SAQ Physical Limitations, SAQ-QL= SAQ Quality of Life. *p* denotes the statistical significance between groups. The values denote adjusted mean changes (95% confidence interval).
